# Does board activeness strengthen the relationship between structure of corporate ownership and firm performance?

**DOI:** 10.3389/fpsyg.2022.1104178

**Published:** 2023-01-10

**Authors:** Feng Yuan, Rana Tanveer Hussain, Iqra Khalid, Mi Li

**Affiliations:** ^1^School of Economics and Management, Jilin Engineering Normal University, Changchun, China; ^2^School of Business and Management Sciences, Minhaj University Lahore, Lahore, Pakistan; ^3^Faculty of Management and Economics, Kunming University of Science and Technology, Kunming, Yunnan, China

**Keywords:** board activeness, firm performance, state ownership, associated ownership, institutional ownership, foreign ownership, family ownership, ownership concentration

## Abstract

This study empirically investigates the moderating effect of board activeness on the relationship between the structure of corporate ownership and firm performance. The objective was evaluated using the hierarchal panel regressions with data from non-financial companies of the Pakistan Stock Exchange from 2009 to 2018, operationalizing the ownership structure as state ownership, associated companies, foreign ownership, ownership concentration, institutional ownership, and family ownership, and firm performance as operating performance, financial performance, and stock market performance. The findings of the study revealed that operating, financial, and stock market performance were favorably influenced by the ownership stakes of the state, associated concerns, institutions, and foreigners. Family interests proved to be diverse for the firm performance. The isolated effect of the board consistently uplifted the firm productivity, but its interactional impact with all the ownership stakeholders postulated differential outcomes for internal and external performance. The study provides valuable insights for policymakers and investors to make optimal strategies to manage ownership interests and enhance value.

## 1. Introduction

Agency expenses and conflicts that may arise between the owners of the organization and executives are reduced by good governance (Alanazi, 2019). Based on various perspectives, “Corporate Governance is the mode through which substances are overseen and represented.” The ownership structure is one of the most important corporate administration factors impacting the extent of agency costs of a firm (Arosa et al., 2010). The distribution of shares among owners has been characterized as the ownership structure in previous studies (Khan et al., 2022), which refers to the stake holdings of families, state, foreign, institutions, managerial, and associates in a growing market (Zureigat, 2015). The opposing perspective on ownership structures focuses on the mix of ownership concentration and control that results from the consequences of the agency dilemma (Fama and Jensen, 1983). This mix permits controlling shareholders to separate private advantages from the firm to the disadvantage of minority shareholders. This inside control system is critical in deciding the destinations of firms, investor riches, and the degree of discipline of managers (Jensen, 2003). The ownership mechanism is equally important to be scaled and measured for the causal effect on the performance of the firm not only in the developed markets but also in emerging ones (Iwasaki et al., 2022). One of the influential mechanisms in reaping the firm performance as per agency mechanism is ownership of possessions by various groups (Alkurdi et al., 2021). Principal-agent mechanism considers each ownership stakeholder as a monitoring force to comply with the primary goal of the firm, i.e., wealth maximization for stockholders. The mechanism of stakeholders supports the finding that resource management in the firm becomes efficient because ownership possessions of stakeholders reduce the expropriation of resources by the managers (Khan and Zahid, 2020). Agency and stakeholder outlooks help firms reduce agency costs. This study deemed these two viewpoints to overcome agency issues in Pakistan.

To cater to governance issues, more than 400 codes have been issued globally since 1992. The Securities and Exchange Commission of Pakistan (SECP) incorporated a code of corporate governance in 2002. This improved the oversight of the board and related matters to enhance the quality of internal control. The 2002 code was further revised in 2012, 2017, and 2019, respectively. The latest codes were gradually strengthened by the inclusion of mandatory provisions concerning board independence, diversity, directorship, respective committees, and/or auditing measures (Khan et al., 2022).

State-owned enterprises expect a fundamental capacity in countries with transitory economies to create integration in the market (Li, 2018). The internationalization of state-owned enterprises has become a major trend in the global economy. State multinationals contribute abroad to get information, innovative and managerial capacities, brands, and other key resources they need (Aleksei, 2018). The job of state-owned elements in a market economy is to advance proficiency in asset allotment (Zhou and Xie, 2016). State-owned enterprises are known by many names, such as government organizations, government business enterprises, government-connected organizations, public enterprises, and public area units (Kenton, 2019). The relationship between company performance and firm valuation was moderated by state ownership (Djankov and Murrell, 2002). Management was believed to be equipped for changing the level of division between the interests of stakeholders (Estiasih et al., 2019). According to the literature, company performance improves with increased ownership, and management interest scheming converges with increased ownership concentration (Katper et al., 2018).

Checking done by the supervisor can influence the movement of the directors of the organization to expand the worth of the organization by comprehensively revealing every one of its resources, and remembering the immaterial resources of the organization for the fiscal reports (Rafaizan et al., 2020). If an administrative proprietorship develops, the director will be bound to further develop execution to help the worth of the organization by uncovering the scholarly capital of the organization. The more prominent the administrative possession, the more intelligent capital is uncovered in the budget summaries (Rafaizan et al., 2020).

The performance and self-serving behavior of managers are assessed by financial performance measures installed by the directors (Katper et al., 2018). Foreign ownership is decidedly identified with greater improvement performance considering the way that new examiners can bring important assets, particularly monetary assets and trendsetting innovations for development exercises (Nguyen-Van and Chang, 2019). Foreign ownership is overall considered to be an important method for acquiring capital, high innovation, and the abilities of the executive to the firm. These assets help to further develop governance and execution (Meng et al., 2018). Possession fixation is seen as a critical corporate administration instrument as owners with concentrated shareholdings sway the exercises and the leaders of an association (Altaf and Shah, 2018). The higher ownership concentration can safeguard minority shareholders from the capture of business visionaries and work on corporate execution, which resembles corporate governance (Kim, 2019). The oversight of the board has a favorable impact on how ownership concentration affects performance (Guerrero-Villegas et al., 2018).

In privately owned companies in emerging economies like Pakistan, one investor from a family owns an enormous part of the stock, and relatives are selected for the chief and functional positions. If the level of chiefs hits a specific level, they might be convinced to introduce a more engaging monetary and execution articulation. Furthermore, managing the behavior of managers allows significant owners to influence the decisions and actions of the firm (Shiri et al., 2018). The variability and temporal fluctuations in the absorptive capacity of the firm can be influenced by family ownership. Family ownership can have an impact on the progressive design and informal social ties of the company, limiting the types of knowledge that can be gained, assimilated, changed, and utilized (Kotlar et al., 2020). All things considered, from an agency perspective, family ownership can decrease review hazard appraisal, lower expenses to support inward checking, and diminish incompatible situations between corporate chiefs and proprietors (Khan and Subramaniam, 2012). Institutional financial backers can assume a fundamental part in checking the organizations, the executives, and their ventures or disinvestments in organizations. Their professional foresight brings prosperity to the firm (Nashier and Gupta, 2016). Institutional financial backers help firm chiefs take advantage of scattered small shareholders (Lin and Fu, 2017).

Keeping the preceding discussion in view, the study aims to determine how the various possessions of stakeholders affect the economic, financial, and stock market execution of non-financial enterprises listed on the Pakistan Stock Exchange. Pakistan is a developing economy with only one national stock market on which a wide range of investors (individuals and institutions, foreign and local, and governmental and non-governmental) can trade and invest in listed companies. The major portions of the stock market concerning the number of firms represent non-financial firms. Being an emerging economy and one of the major economic indicators, the stock market is very attractive for generating returns in the region. However, at the same time, it is considered a very volatile market. Therefore, different stakeholders and ownership perspectives want to protect their wealth. This makes it evident to conduct such a study and gives hints to the stakeholders on how to save their interest by using the effect of ownership structures on firm execution. Another important point is that this is also going to become empirical evidence by highlighting the supervisory role of the board. The more the board is active, the more it can play a significant role in creating harmony between the expectations of stakeholders and firm execution. Various studies (Jabeen and Ali, 2017; Farooq and Manzoor, 2019; Usman and Alam, 2020; Hussain et al., 2022) have been done on the relationship between ownership and performance in Pakistan. They gave empirical evidence on ownership studies and asserted that ownership has become a prominent tool to gauge the performance of the firm. The agency problem from the ownership context has not yet been properly addressed in the Pakistani context because of the limited scope taken in the previous studies, especially when various stakeholders become part of ownership. Keeping in view the context of agency theory and stakeholder theory, Khan et al. (2022) recommended a comprehensive study on ownership formation to address the agency issue. Additionally, board insertion at the time of pursuing the respective interests of each stakeholder was also required to be addressed. This study responded to both by conducting a comprehensive framework on ownership, possession, and board activeness and focusing on agency issues.

Researchers, investors, practitioners, and politicians can all benefit from this study. The research gives decision-makers and practitioners in-depth knowledge about the risks connected with the state, related concerns, foreign, ownership concentration, and institutional and family ownership on business execution in terms of non-financial firms. The findings of the study are anticipated to be helpful to investors in their investment decisions, particularly regarding ownership by the government, related businesses, institutions, and foreigners. First, the management of the firm should specifically use the state entry in the ownership. The policies of the firm should be in accordance with Pakistani government laws and regulations. As a mentor, the state finances the use of the resources by firms. The firm is more secure as it is closer to the state. Second, the study showed how helpful associated companies are to the business. The company should use the purchasing of associates, selling, and credit services. Third, the results of the numerous studies consistently show that institutions in the ownership act as guardian angels of a firm, not only by providing financial support but also by addressing problems with the agency. Management can illuminate its credit darkness by the optimal linkage with the institutions. Fourth, the strength of the overseas portfolios provides a very obvious signal that the management of the firm should use foreign holdings to access international markets. Fifth, management must look out for the interests of the family and key stakeholders. When there are conflicting results, the role of other stakeholders becomes crucial. Sixth, efficient boards get rid of all the obstacles to increased performance. They manage the harmful externality that exists between agents and principles. Seventh, when the board interacts with stakeholders, an effective management plan is required. Market participants have a huge impact on the Pakistani market. To maintain the interest of everyone, it is important to closely monitor the varied outcomes of the interactions of stakeholders with the board.

The article is structured as follows: after the preliminary discussion in the section “1 Introduction,” literature regarding the causal relations of the study and their respective hypotheses are outlined in the section “2 Literature review and hypothesis development.” Research methodology explained the data, methods, techniques, and variables in the section “3 Research methodology.” A causal relation is examined in the section “4 Results and discussion,” followed by the conclusion of the study.

## 2. Literature review and hypothesis development

The agency theory, according to Eisenhardt (1989), is concerned with the universal agency connection, in which the principle assigns tasks to the agent. In terms of business organizations, the agency theory entails a contract in which the shareholders engage the management to provide some function on their behalf, including assigning certain decision-making authority to the managers (Jensen and Meckling, 1976). The stakeholder theory is a hypothesis about the connection of a company with its stakeholders. Shareholders, creditors, employees, public interest groups, customers, suppliers, governmental agencies, and the community are among the stakeholders identified as having a stake in a firm and having something at risk (Chiu and Wang, 2015). Shao (2019) provided a wide-growing assessment of the association between corporate administration plans and firm execution in Chinese recorded firms from 2001 to 2015. Ownership positions suggested differential performance execution. Buallay et al. (2017) respond to the question, “Is there any connection between CG and firm execution?” The ownership structure was found significant for the performance. Mashayekhi and Bazaz (2008) showed that the presence of institutional financial backers on the top managerial staff was decidedly connected with firm execution. Zraiq and Fadzil (2018) achieved the essential objective by analyzing the connection between ownership development and firm execution of the Jordanian firms. Their discoveries demonstrated an altogether sure connection between possession structure (family and other) and firm execution. Kapopoulos and Lazaretou (2007) explored that a more careful proprietorship structure decidedly connects with higher firm productivity.

### 2.1. State ownership and firm performance

Diversified contexts showing both positive and negative influence of state ownership have been observed in the empirical literature. Iwasaki et al. (2022) discovered the 4,425 outcomes from 204 studies that were conducted in China, Russia, and EU states. They argued about the adverse effect of the state on firm progress. The same harmful impact was reported by Amin and Haq (2022) in Russia, China, and India. Earlier, Queiri et al. (2021) also found the same injurious effect of state ownership on firm performance. The reason for the negative effects of the state-owned enterprise was that state enterprises had been considered common property, which is named the “tragedy of the common,” where utilities of various stakeholders are linked to such firms for their interests. In contrast, the state also brought positivity to the firm. Boubakri et al. (2020) and Aguilera et al. (2021) demonstrated the positive contribution of government ownership in the development and growth of the firm. Diverse stakeholder interests in the ownership lead to a conflict of interest and an agency problem. However, according to stakeholder theory, this varied interest is also the source of pressure to undermine the negativity of the state mechanism. Agency theory suggested that firms act as agents of the state by following regulations and policies (Liu et al., 2020). To keep the principal–agent relationship intact, the board of the firm can play an effective role in minimizing the negative effect of state possession and saving agency costs and political costs (Iwasaki et al., 2022). The institutional theory contends that the actions of firms get shaped by the environment in which they operate. Institutions like states legitimize the policies, laws, and regulations under which firms operate. The prosperity of a firm depends upon compliance with the regulations drawn by the state. State-owned firms brought positivity to the environment (Wang and Jiang, 2021). The following hypothesis is proposed:

*Hypothesis 1: State ownership has a positive effect on firm execution.*

### 2.2. Associated ownership and firm performance

Associations provide opportunities for the firm to achieve better performance. Farooq et al. (2020) asserted negative associations between associated ownership and distress, which leads to value addition. Managerial ownership had an immediate and backhanded adverse consequence on firm worth through scholarly capital as a mediating variable, and scholarly capital negatively affects firm worth (Rafaizan et al., 2020). Waemustafa (2018) depicted a non-linear relationship between both cash holding and capital development decisions of material firms. Their results similarly offered assistance to association speculation, pecking order theory, and hailing speculation. Yusra et al. (2019) used board information as a relapse strategy. The administrative possession essentially and emphatically influences the worth of the association. Cui and Mak (2002) checked out that Tobin’s Q at first decreased with an administrative proprietorship. Their discoveries recommend that industry impacts are significant in the connection between administrative proprietorship and the achievement of associates. The executive of the company is the shareholder of the company share. Managerial proprietorship is perceived to be good for the firm performance. Firm performance gets improved when ownership and managerial interests are merged, and the following hypothesis is proposed:

*Hypothesis 2: Associated ownership has a positive effect on firm execution.*

### 2.3. Institutional ownership and firm performance

Agency theory and stakeholder perspective considered financial institutions as building pressure on the agents to run the firm on the value-generating path. The institutions, after taking ownership stakes, inserted a positive effect on financial and market areas of performance (Alkurdi et al., 2021). The same influence was depicted in the studies of Drobetz et al. (2021) and Saleh et al. (2022). The internal and external performance of the firm was positively influenced by the local and international institutional owners (Abedin et al., 2022). Lin and Fu (2017), from 2004 to 2014, examined that institutional ownership decidedly influences firm performance and is powerful to represent liberation, contemporaneous economic situations, and diverse security exchanges. Specifically, the outcomes showed that pressure-inhumane, foreign, and huge institutional investors have more noteworthy beneficial outcomes on firm execution than pressure-touchy, homegrown, and less institutional investors. Nashier and Gupta (2020) disclosed that institutional possession emphatically affects firm execution. According to Thanatawee (2014), esteem ownership by a local institutional monetary sponsor unequivocally influences firm advantages and higher new institutional belonging is connected with lower corporate worth. According to a review by Handriani and Robiyanto (2019) of institutional belonging, the main gathering of independence has a beneficial outcome simply on Tobin’s Q regard, whereas the board size can grow Tobin’s Q. Rong et al. (2017) found that the presence of institutional monetary patrons works on firm headway. The corporate governing functions of institutional owners can reduce agency problems and improve firm performance. Institutional investors can affect the management activities directly as owners of firms or indirectly through trading in securities of such firms. The following hypothesis is proposed:

*Hypothesis 3: Institutional ownership has a positive effect on firm execution.*

### 2.4. Foreign ownership and firm performance

From the perspective of stakeholders, foreigners carefully monitor the executives, which leads to value creation for the firm (Iwasaki et al., 2022). Similar positive insertions on accounting and market-based performance by the foreign owners were depicted by Rashid (2020) and Din et al. (2021). Duong et al. (2021) discovered a U-shaped relationship between foreign ownership and firm outcomes. Performance of the firm gave a positive response to foreign ownership up to 32.26% but afterward, declined. In line with the agency perspective, after comparing the high and low-performing firms, Ha and Tran (2021) proclaimed that the impact of the foreign stake was pronounced more affirmative in larger performers. Jusoh (2016) analyzed that foreign ownership had a favorable and strong association with ROA and Tobin’s Q. It minimizes agency conflict. Azzam et al. (2013), comparing the impact of different levels of foreign possession on monetary execution, examined that foreign proprietorship increases monetary execution up to a level and afterward decreases. Their data suggested that foreign ownership had a sector-specific impact. Foreign equity investment encouraged domestic enterprises to innovate. Foreign proprietorship positively affected the advancement exercises of firms. Foreign ownership encouraged innovation through forward linkage, and this effect is even stronger in chaebol firms (Joe et al., 2019). Koch and Smolka (2019) provided novel confirmation that firms with foreign aid viably raised the aptitudes of their labor force by enlisting high-skilled workers and giving them expert readiness. da Silva et al. (2018) asserted that foreign ownership was positively related to more innovation performance because foreign investors can bring necessary resources, especially financial resources, and advanced technologies for innovation activities. The following hypothesis is proposed:

*Hypothesis 4: Foreign ownership has a positive effect on firm execution.*

### 2.5. Family ownership and firm performance

Controversy existed about whether family stakes in ownership moved the performance in a positive or negative direction. The family business contributed to a large extent to the national economy (Jadoon et al., 2021). From the agency perspective, family ownership was pronounced unfavorable for the firm (Amin et al., 2022). However, measuring the Italian market behavior, Pierni et al. (2022) asserted that performance peaked in family founded firms. Minh Ha et al. (2022) presented interesting findings in the context of Vietnam. Performance was non-linearly related to family ownership. Accounting and market performance changed the direction after 65.89 and 42.53% ownership stake by families. Srivastava and Bhatia (2022) also depicted a U-shaped relationship. As a stakeholder, the family drives the performance toward value addition. When the state and family as owners interacted with each other, the performance of the firm increased (Martínez-García et al., 2021). Subramaniam (2018) looked through the results, considering that family ownership apparently had a colossal positive relationship with firm benefits in Malaysia, especially. In contrast, Kim et al. (2017), according to an observational study, posited that Korean family ownership reduced the value of a company when such controlling shareholders participated in the management and pursued excessive pay or when management entrenchment effects were related to ownership-control discrepancies. The agency costs associated with obtaining increased executive compensation or private benefits lowered firm value when controlling owners of family enterprises had expanded control rights over the general meeting of shareholders and the board of directors. Chu (2011) found that family ownership was insistently associated with firm execution. The positive association was strong, particularly when family members were filled in by CEOs, top bosses, chiefs, or regulators of the associations. The following hypothesis is proposed:

*Hypothesis 5: Family ownership has a positive effect on firm execution*.

### 2.6. Ownership concentration and firm performance

Considering the agency view, ownership concentration reduced the asymmetry of information, which led to a favorable outcome for the firm (Javeed et al., 2021). The proponent of the agency perspective considered the concentration as an effective tool of monitoring in favor of the firm, while opponents of the theory considered it in opposite direction (Shahrier et al., 2020). Firms generated valuable outcomes by utilizing the ownership possessed by the top leaders (Chatterjee and Bhattacharjee, 2021; Din et al., 2021; Javeed et al., 2021). Some studies revealed that financial and market operations were inversely caused by concentrated ownership (Alkurdi et al., 2021; Martínez-García et al., 2021; Queiri et al., 2021). Ownership concentration is a significant internal corporate governance mechanism through which owners can control and influence the management of the firm to protect their interests. Nashier and Gupta (2020) portrayed ownership concentration as a huge corporate governance system that impacted the tasks and executions of Indian organizations. They observed that ownership concentration decidedly influenced both the market and bookkeeping performance of an organization. Their outcomes recommended that concentrated ownership diminished agency costs as block holders effectively screened the administration of the organization, consequently prompting better firm performance. Guerrero-Villegas et al. (2018) monitored that the checking given by the board unequivocally impacted the effect that belonging obsession had on execution. Anwar and Tabassum (2011) recommended that there was a huge positive relationship between possession fixation and the working execution of companies. The following hypothesis is proposed:

*Hypothesis 6: Ownership concentration has a consequential effect on firm execution.*

### 2.7. Board activeness and firm performance

Ali et al. (2022) asserted the significance of the multifunctionality of the board in reducing the distress situation of the firm. The board diversity benefits the firm during the interim meetings. Frequent board meetings are very important for the performance of any firm. They showed attentiveness to the board. Frequent board meetings refer to the diligence and ability of the board to perform regular monitoring and advisory services for the managers of the firms. Salim et al. (2016) showed that the performance of Australian banks was better than their fellow firms due to frequent board meetings, which improved the solvency of the firm. Andreou et al. (2014) also found that the number of board meetings was strongly correlated with financial management decisions and firm performance. Activities done by the board in the form of conducting meetings for the strategic assessment and financial disclosure of the firm imply the diligence of the board. The frequency of meetings had a positive relationship with the probability of distress (Khurshid et al., 2018). Various studies (Al-Musali and Ismail, 2015; Dakhlallh et al., 2019; Bendig et al., 2020; Al-Qatanani and Siam, 2021) highlighted the moderating role of the board from various perspectives of the firm. An effective board is expected to avoid politics and enhance managerial accountability to safeguard the interests of key stakeholders. The board not only oversees the agency issues but also resolves them effectively if raised among stakeholders (Queiri et al., 2021). Puni and Anlesinya (2020) recommended the role of the board in adding value to the firm. The inverse impact is also presented for block ownership and firm execution (Queiri et al., 2021). The board plays a significant role in moderating the ownership parameters and performance. Therefore, it can be hypothesized as follows:

*Hypothesis 7: The high activeness of the board strengthens the firm performance.*

*Hypothesis 8: Board activeness asserts a moderating effect between ownership structure and firm performance.*

## 3. Research methodology

This study was explanatory in nature based on the quantitative method being conducted on listed companies of the Pakistan Stock Exchange by choosing non-financial firms as a sample study. The data for the post-global financial period (i.e., 2009–2018) were manually extracted from the audited annual financial reports of non-financial listed firms in the Pakistan Stock Bourse (PSX). These reports have been submitted to the SECP and are also available on the official websites of the PSX and the concerned listed firm. The explained factors were as follows in Table 1: operating performance was measured by total income to total sales (Hussain and Waheed, 2019). Financial performance was measured by net income to total assets (Hussain and Waheed, 2019). The market-to-equity ratio was measured by the market value per share to book value per share (Hussain and Waheed, 2019). Explanatory factors were as follows: state ownership was measured by the total shares owned by the state to the total number of shares (Vu and Pratoomsuwan, 2019). Foreign ownership was measured by the percentage of foreign investor shares to total shares (Ciftci et al., 2019). Associated companies were measured as the percentage of shares held by associated companies (Shao, 2019). Ownership concentration was measured by the largest ten shareholding percentages and the largest five shareholding percentages (Shao, 2019). Institutional ownership was measured by the percentage of the number of shares owned by the institution to the total number of shares outstanding (Handriani and Robiyanto, 2019). Family ownership was measured by the percentage of shares owned by the founders and their family members over the total shareholdings in the firm (Subramaniam, 2018). Board activeness was used as a moderating aspect and was measured by the number of annual meetings of the board of directors (Vitolla et al., 2020). Control factors were the firm size, age, and leverage. The natural logarithm of total assets was used to calculate the firm size (Ullah et al., 2017). The firm age was calculated using the natural log of the firm age from the date of incorporation (Ciftci et al., 2019). The firm leverage was calculated as total debt/total equity (Shao, 2019).

**TABLE 1:** Measurement of variables.

Variables	Measurements	References
Operating performance (OPER)	Net income/total sales	Hussain and Waheed, 2019
Financial performance (FPER)	Net income/total asset	Hussain and Waheed, 2019
Market to equity ratio (MPER)	The market value per share/book value per share	Hussain and Waheed, 2019
State ownership (SOWN)	Total shares owned by state to total number of shares	Vu and Pratoomsuwan, 2019
Foreign ownership (FOWN)	Percentage of foreign investor shares to total shares	Ciftci et al., 2019
Associated companies ownership (ASOWN)	The percentage of shares held by associated companies	Shao, 2019
Ownership concentration (OWNC)	The largest ten shareholding percentages and the largest five shareholding percentages	Shao, 2019
Institutional ownership (IOWN)	The percentage of the number of shares owned by the institution to the total number of shares outstanding	Handriani and Robiyanto, 2019
Family ownership (FAMOWN)	The percentage of share ownership of the founders and their family members over the total shareholdings in the firm	Subramaniam, 2018
Board activeness (BAACTIVE)	The number of annual meetings of the board of directors	Vitolla et al., 2020
Firm size (SIZE)	Natural logarithm of total assets	Ullah et al., 2017
Firm age (AGE)	Natural log of age of firm from date of incorporation	Ciftci et al., 2019
Firm leverage (LEV)	Total debt/total equity	Shao, 2019

The following multiple regression models was built to explore the moderating influence of board activeness on the relationship between corporate ownership structure and firm execution for all non-financial firms listed on the Pakistan Stock Exchange during the period 2009–2018. Following the panel regression model, the proposed relation was measured where performance, as an explained variable, was operationalized by operating, financial, and stock market performances.


$$\begin{array}{l} Performance_{i,t}\\ =\beta_{0}+\beta_{1}SOWN_{\text{it}}+\beta_{2}ASOWN_{\text{it}}+\beta_{3}IOWN_{\text{it}}\\ \;\;+\beta_{4}FOWN_{\text{it}}+\beta_{5}FAMOWN_{\text{it}}+\beta_{6}OWNC_{\text{it}}\\ \;\;+\beta_{7}BACTIVE_{\text{it}}+\beta_{8}(SOWN_{\text{it}}*ACTIVE_{\text{it}})\\ \;\;+\beta_{9}(ASOWN_{\text{it}}*ACTIVE_{\text{it}})+\beta_{10}(IOWN_{\text{it}}\\ \;\;*ACTIVE_{\text{it}})+\beta_{11}(FOWN_{\text{it}}*ACTIVE_{\text{it}})+\beta_{12}\\ \;\;\;(FAMOWN_{\text{it}}*ACTIVE_{\text{it}})+\beta_{13}(OWNC_{\text{it}}*ACTIVE_{\text{it}})\\ \;\;+\beta_{14}SIZE_{\text{it}}+\beta_{15}AGE_{\text{it}}+\beta_{16}LEV_{\text{it}}+e_{\text{it}} \end{array}$$

## 4. Results and discussion

The descriptive analysis, as shown in Table 2, provides insight into the behavior of the variable. The average performance of the operating, financial, and stock market perspectives of the firms was in positive zones, but their deviation was greater than their average performances. The minimum and maximum ranges showed wide deviation values, which depict the volatility in the profitability of the non-financial firms of Pakistan. The same is true for the other variables of the study, especially the explanatory factors. The state owned 2.07% stakes in the firms, which increased to 85.25%. Associated companies owned 27.53%, ranging to 98.02%. Institutions held 21.9% stakes in the firms, with a maximum of 73.4%. Foreigners acquired 3.06% of shares, with a maximum holding of 76.91%. The average holding of the families of the firms was 17.82%, with a maximum stake of 97.71%. The average value of the ownership concentration in the hands of the top five holders was 60.82. The average number of meetings held during the period was 5, with a maximum of 19.

**TABLE 2:** Summary statistics.

	*N*	Mean	Std. Dev.	Min	Max
OPER	1,683	1.76	38.46	−271.73	123.38
FPER	1,708	14.13	39.15	−14.39	175.00
MPER	1,643	2.95	6.35	0.00	27.24
SOWN	1,752	2.07	10.61	0.00	85.25
ASOWN	1,752	27.53	30.37	0.00	98.02
IOWN	1,752	21.90	19.71	0.65	73.44
FOWN	1,752	3.06	11.46	0.00	76.91
FAMOWN	1,752	17.82	25.19	0.00	97.71
OWNC	1,679	60.85	22.85	16.33	96.65
BACTIVE	1,656	5.45	1.33	3.00	19.00
SIZE	1,709	14.80	2.49	5.66	19.95
AGE	1,750	38.83	26.89	4	203
LEV	1,699	1.255	0.9342	0.2232	2.65

Table 3 demonstrates the relationship. The relationship between the operating, financial, and stock market performing areas of the firms and the explanatory factors was mixed. Among all the explanatory variables, institutional ownership and family ownership had a negative relationship with operating performance. Ownership of the state-associated companies, foreigners, ownership concentration and board activeness showed a negative association with financial performance. Ownership of the state, institutions, and families and ownership concentration showed a negative association with the stock market. Pairwise correlation among the explanatory variables did not posit any issue of collinearity; however, the multicollinearity test was separately run, which asserted that multicollinearity was not a problem in all panel regressions. The Breusch–Pagan test was run to test the issue of heteroskedasticity. The outcome of the test depicted heteroscedasticity issues in almost all the models. To cover this problem, panel regression analysis with robust standard errors was applied to all models to explain the results. Various econometric techniques are used to evaluate the influence of a cause over effect. After diagnosing the multiple linear regression assumptions, optimal estimates were extracted using the panel OLS method (Abedin et al., 2022; Amin and Haq, 2022; Pierni et al., 2022).

**TABLE 3:** Pairwise correlations.

Variables	(1)	(2)	(3)	(4)	(5)	(6)	(7)	(8)	(9)	(10)	(11)	(12)	(13)	VIF
(1) OPER	1.000													
(2) FPER	0.010	1.000												
(3) MPER	0.001	−0.003	1.000											
(4) SOWN	0.003	−0.016	−0.015	1.000										1.26
(5) ASOWN	0.018	−0.065*	0.114*	−0.145*	1.000									1.81
(6) IOWN	−0.009	0.026	−0.008	−0.032	−0.210*	1.000								1.41
(7) FOWN	0.001	−0.017	0.017	0.173*	−0.126*	−0.056*	1.000							1.22
(8) FAMOWN	−0.029	0.012	−0.054*	−0.111*	−0.412*	−0.220*	−0.134*	1.000						1.70
(9) OWNC	0.001	−0.005	−0.005	0.165*	−0.040	0.075*	0.002	−0.044	1.000					1.05
(10) BACTIVE	0.029	−0.047	0.081*	0.106*	0.043	0.022	0.006	−0.038	0.025	1.000				1.05
(11) SIZE	0.018	−0.234*	−0.203*	0.151*	0.163*	−0.006	0.072*	−0.194*	0.110*	0.065*	1.000			2.05
(12) AGE	−0.009	0.002	0.068*	−0.011	−0.058*	−0.056*	0.024	−0.019	0.010	−0.023	−0.003	1.000		1.16
(13) LEV	0.001	0.000	0.009	0.012	−0.004	0.013	0.012	−0.013	0.018	0.030	−0.030	0.008	1.000	1.10

*p < 0.1.

Panel regression analysis of all performing parameters (operating, financial, and stock market) is described in Tables 4–6, respectively. All the tables describe the results of panel regressions with robust standard errors to overcome the heteroskedasticity issue. State ownership (SOWN) inferred a positive influence on the operating outcomes of the firms. The assertion was consistent with the financial and stock market performing areas and was also aligned with Eforis (2018), Boubakri et al. (2020), Aguilera et al. (2021), and Wang and Jiang (2021). It supported the first hypothesis. The emerging economy of Pakistan gets a fruitful insight from this result that state ownership improves the productivity of the firm. Associated companies (ASOWN) influenced the operating, financial, and stock market outcomes. The significance of the result supported the second hypothesis and the study of Rafaizan et al. (2020). The consistency of the results recommends the associated companies as stakeholders of the firm. The operational, financial, and stock market performance were also impacted affirmatively by institutional ownership (IOWN). The finding of this study with respect to institutional ownership was in support of the third hypothesis and in line with Nashier and Gupta (2020), Alkurdi et al. (2021), Drobetz et al. (2021), and Saleh et al. (2022), revealing that institutional ownership has an efficacious force on firm execution. This outcome has various theoretical aspects, such as agency theory and resource dependency theory.

**TABLE 4:** Regression analysis of operating performance.

	(1)	(2)	(3)	(4)	(5)	(6)	(7)	(8)
	LOPER	LOPER	LOPER	LOPER	LOPER	LOPER	LOPER	LOPER
SOWN	0.0096***	0.0519***	0.0095***	0.0095***	0.0104***	0.0096***	0.0091***	0.0591***
	(0.0035)	(0.0083)	(0.0035)	(0.0035)	(0.0036)	(0.0035)	(0.0035)	(0.0112)
ASOWN	0.0008	0.0008	0.003	0.0008	0.0008	0.0008	0.0008	0.0158*
	(0.0017)	(0.0017)	(0.0065)	(0.0017)	(0.0017)	(0.0017)	(0.0017)	(0.0082)
IOWN	0.0088***	0.0086***	0.0088***	0.0133	0.0088***	0.0088***	0.0089***	0.027***
	(0.002)	(0.002)	(0.002)	(0.0081)	(0.002)	(0.002)	(0.002)	(0.0098)
FOWN	0.0076*	0.0084**	0.0076*	0.0077*	0.0224	0.0077*	0.0075*	0.0234
	(0.0041)	(0.0041)	(0.0041)	(0.0041)	(0.0175)	(0.0041)	(0.0041)	(0.0189)
FAMOWN	−0.0038*	−0.0037*	−0.0038*	−0.0038*	−0.0038*	−0.0026	−0.0038*	0.0115
	(0.002)	(0.002)	(0.002)	(0.002)	(0.002)	(0.0068)	(0.002)	(0.0084)
OWNC	−0.0001**	−0.0001**	−0.0001**	−0.0001**	−0.0001**	−0.0001**	0.0035*	0.0029
	(0.00)	(0.00)	(0.00)	(0.00)	(0.00)	(0.00)	(0.0021)	(0.0022)
BACTIVE	0.0383	0.0554	0.0493	0.0543	0.0446	0.0432	0.0817*	0.3069***
	(0.0353)	(0.0371)	(0.0449)	(0.0518)	(0.0366)	(0.0454)	(0.0426)	(0.1106)
SIZE	−0.0461***	−0.0413***	−0.0461***	−0.0465***	−0.0466***	−0.046***	−0.0464***	−0.042***
	(0.0151)	(0.0151)	(0.0151)	(0.0151)	(0.015)	(0.0151)	(0.015)	(0.0152)
AGE	−0.0003	−0.0001	−0.0002	−0.0003	−0.0005	−0.0003	−0.0003	−0.0002
	(0.0013)	(0.0013)	(0.0013)	(0.0013)	(0.0014)	(0.0013)	(0.0013)	(0.0014)
LEV	0.002***	0.002***	0.002***	0.002***	0.002***	0.002***	0.002***	0.002***
	(0.0002)	(0.0002)	(0.0002)	(0.0002)	(0.0002)	(0.0002)	(0.0002)	(0.0002)
SOWN × BA		−0.007***						−0.0084***
		(0.0017)						(0.0021)
ASOWN × BA			−0.0004					−0.0028*
			(0.0011)					(0.0015)
IOWN × BA				−0.0008				−0.0033*
				(0.0015)				(0.0018)
FOWN × BA					−0.0028			−0.0028
					(0.0031)			(0.0034)
FAMOWN × BA						−0.0002		−0.0028*
						(0.0012)		(0.0015)
OWNC × BA							−0.0006*	−0.0005
							(0.0003)	(0.0004)
_cons	1.9525***	1.7839***	1.8908***	1.8721***	1.9357***	1.9243***	1.6985***	0.4068
	(0.3133)	(0.3249)	(0.3443)	(0.3547)	(0.3161)	(0.3591)	(0.3384)	(0.6338)
Observations	1196	1196	1196	1196	1196	1196	1196	1196
R-squared	0.0624	0.0681	0.0625	0.0626	0.0629	0.0624	0.0644	0.0736

Robust standard errors are in parentheses. LOPER means a log of the operating performance of the firm. ***p < 0.01, **p < 0.05, *p < 0.1. LOPER is the log of operating performance.

SOWN is the state ownership. ASOWN is the ownership-associated firm. IOWN is the institutional ownership. FOWN is foreign ownership. FAMOWN is family ownership. OWNC is ownership of top block holders. BACTIVE is the number of meetings. SOWN × BA, ASOWN × BA, IOWN × BA, FOWN × BA, FAMOWN × BA, and OWNC × BA are the interactional relationships of ownership stakes with board activity. SIZE is the log of total assets. AGE is the age of the firm since its inception. LEV is the ratio of debt to total assets.

**TABLE 5:** Regression analysis of financial performance.

	(1)	(2)	(3)	(4)	(5)	(6)	(7)	(8)
	LFPER	LFPER	LFPER	LFPER	LFPER	LFPER	LFPER	LFPER
SOWN	0.0211***	0.0022	0.0214***	0.0207***	0.0227***	0.0207***	0.0205***	0.001
	(0.003)	(0.0085)	(0.003)	(0.003)	(0.0029)	(0.0029)	(0.0031)	(0.0124)
ASOWN	0.0039*	0.004*	−0.0025	0.004*	0.0039*	0.0039*	0.0039*	0.0101
	(0.002)	(0.002)	(0.008)	(0.002)	(0.002)	(0.002)	(0.002)	(0.01)
IOWN	0.0081***	0.0082***	0.0082***	0.0241**	0.008***	0.0082***	0.0082***	0.037***
	(0.0028)	(0.0028)	(0.0028)	(0.0097)	(0.0028)	(0.0028)	(0.0028)	(0.0106)
FOWN	0.0098**	0.0095**	0.01**	0.01**	0.0404*	0.01**	0.0097**	0.0616***
	(0.0042)	(0.0042)	(0.0042)	(0.0042)	(0.021)	(0.0042)	(0.0042)	(0.0218)
FAMOWN	0.005**	0.0049**	0.005**	0.0051**	0.0049**	0.0157**	0.0049**	0.0248***
	(0.0024)	(0.0024)	(0.0024)	(0.0024)	(0.0024)	(0.0065)	(0.0024)	(0.0081)
OWNC	0.001	0.001	0.001	0.001	0.001	0.001	0.004	0.0055*
	(0.003)	(0.003)	(0.003)	(0.003)	(0.003)	(0.003)	(0.0031)	(0.003)
BACTIVE	0.0096	0.0022	−0.0223	0.0657	0.0225	0.0569	0.0585	0.307***
	(0.0399)	(0.0407)	(0.0493)	(0.0545)	(0.042)	(0.0513)	(0.0527)	(0.1094)
SIZE	−0.5049***	−0.507***	−0.5049***	−0.5063***	−0.5059***	−0.5044***	−0.5053***	−0.511***
	(0.033)	(0.0334)	(0.033)	(0.033)	(0.0331)	(0.0329)	(0.033)	(0.0336)
AGE	0.0033	0.0033	0.0032	0.0031	0.0028	0.0033	0.0033	0.002
	(0.0024)	(0.0024)	(0.0023)	(0.0023)	(0.0024)	(0.0023)	(0.0024)	(0.0024)
LEV	0.0013***	0.0013***	0.0013***	0.0013***	0.0013***	0.0013***	0.0013***	0.0013***
	(0.0002)	(0.0002)	(0.0002)	(0.0002)	(0.0002)	(0.0002)	(0.0002)	(0.0002)
SOWN × BA		0.0031**						0.0035
		(0.0015)						(0.0021)
ASOWN × BA			0.0012					−0.0011
			(0.0014)					(0.0018)
IOWN × BA				−0.0029*				−0.0052***
				(0.0017)				(0.0019)
FOWN × BA					−0.0058			−0.0098**
					(0.0038)			(0.004)
FAMOW × BA						−0.002*		−0.0036**
						(0.0011)		(0.0014)
OWNC × BA							−0.0007	−0.0009*
							(0.0005)	(0.0005)
_cons	9.0194***	9.0938***	9.1972***	8.7378***	8.9863***	8.7514***	8.7334***	7.4925***
	(0.5504)	(0.5673)	(0.5886)	(0.5621)	(0.552)	(0.5679)	(0.5887)	(0.7805)
Observations	1200	1200	1200	1200	1200	1200	1200	1200
R-squared	0.3573	0.3578	0.3577	0.3585	0.3581	0.3585	0.3584	0.3648

Robust standard errors are in parentheses. ***p < 0.01, **p < 0.05, *p < 0.1. LFPER is the log of financial performance. SOWN is the state ownership. ASOWN is the ownership-associated firm. IOWN is the institutional ownership. FOWN is the foreign ownership. FAMOWN is the family ownership. OWNC is ownership of top block holders. BACTIVE is the number of meetings. SOWN × BA, ASOWN × BA, IOWN × BA, FOWN × BA, FAMOWN × BA, and OWNC × BA are the interactional relationships of ownership stakes with board activity.

SIZE is the log of total assets. AGE is the age of the firms since inception. LEV is the ratio of debt to total assets.

**TABLE 6:** Regression analysis of stock market performance.

	(1)	(2)	(3)	(4)	(5)	(6)	(7)	(8)
	LMPER	LMPER	LMPER	LMPER	LMPER	LMPER	LMPER	LMPER
SOWN	0.0292***	0.1077***	0.0304***	0.029***	0.0357***	0.0287***	0.0307***	0.1085***
	(0.0049)	(0.0256)	(0.0048)	(0.0049)	(0.0045)	(0.005)	(0.0047)	(0.0161)
ASOWN	0.0224***	0.0224***	−0.0039	0.0224***	0.0221***	0.0224***	0.0225***	0.0098
	(0.0022)	(0.0022)	(0.009)	(0.0022)	(0.0021)	(0.0022)	(0.0022)	(0.0108)
IOWN	0.0174***	0.0171***	0.0177***	0.0284***	0.0167***	0.0175***	0.0173***	0.0271**
	(0.0028)	(0.0028)	(0.0028)	(0.011)	(0.0028)	(0.0028)	(0.0028)	(0.0114)
FOWN	0.0218***	0.0236***	0.0226***	0.0217***	0.1439***	0.0219***	0.0223***	0.1451***
	(0.005)	(0.005)	(0.005)	(0.0051)	(0.0277)	(0.0051)	(0.005)	(0.0289)
FAMOWN	0.0028	0.003	0.0029	0.003	0.0029	0.0124	0.003	0.0155
	(0.0023)	(0.0023)	(0.0023)	(0.0023)	(0.0023)	(0.0086)	(0.0023)	(0.0096)
OWNC	0.001	0.001	0.001	0.001	0.001	0.001	−0.0099**	−0.0115***
	(0)	(0)	(0)	(0)	(0)	(0)	(0.0041)	(0.0044)
BACTIVE	0.2101***	0.236***	0.0849	0.247***	0.2593***	0.2555***	0.095	0.1827*
	(0.049)	(0.051)	(0.054)	(0.0616)	(0.0532)	(0.0615)	(0.0641)	(0.1074)
SIZE	−0.362***	−0.3551***	−0.3591***	−0.3626***	−0.3656***	−0.3616***	−0.361***	−0.357***
	(0.0327)	(0.0328)	(0.0323)	(0.0327)	(0.0323)	(0.0327)	(0.0327)	(0.0322)
AGE	0.0028	0.0029	0.0026	0.0028	0.0024	0.0028	0.0028	0.0024
	(0.0027)	(0.0027)	(0.0026)	(0.0027)	(0.0024)	(0.0027)	(0.0027)	(0.0024)
LEV	0.0475***	0.0486***	0.0459***	0.0468***	0.0513***	0.0477***	0.0465***	0.0501***
	(0.0115)	(0.0118)	(0.0113)	(0.0115)	(0.0129)	(0.0115)	(0.011)	(0.0124)
SOWN × BA		−0.0129***						−0.0117***
		(0.0039)						(0.0026)
ASOWN × BA			0.0048***					0.0022
			(0.0016)					(0.002)
IOWN × BA				−0.002				−0.0019
				(0.002)				(0.0021)
FOWN × BA					−0.0224***			−0.0222***
					(0.0045)			(0.0048)
FAMOWN × BA						−0.0018		−0.0022
						(0.0015)		(0.0017)
OWNC × BA							0.0016**	0.0019***
							(0.0007)	(0.0007)
_cons	2.8824***	2.6321***	3.5201***	2.6863***	2.6844***	2.6252***	3.557***	3.0298***
	(0.5138)	(0.5255)	(0.5483)	(0.5387)	(0.5084)	(0.5401)	(0.5763)	(0.7143)
Observations	1446	1446	1446	1446	1446	1446	1446	1446
R-squared	0.242	0.2489	0.2498	0.2426	0.2568	0.243	0.2474	0.2746

Robust standard errors are in parentheses. ***p < 0.01, **p < 0.05, *p < 0.1. LMPER is the log of stock market performance. SOWN is the state ownership. ASOWN is the ownership associated firms. IOWN is the institutional ownership. FOWN is the foreign ownership. FAMOWN is family ownership. OWNC is ownership of top block holders. BACTIVE is the number of meetings. SOWN × BA, ASOWN × BA, IOWN × BA, FOWN × BA, FAMOWN × BA, and OWNC × BA are the interactional relationships of ownership stakes with board activity. SIZE is the log of total assets. AGE is the age of the firms since inception. LEV is the ratio of debt to total assets.

Foreign ownership (FOWN) also sent a positive signal to the operating, financial, and stock market areas. Firms improved their performances with foreign stakes. The finding of this study with respect to foreign ownership is consistent with Jusoh (2016), Rashid (2020), Din et al. (2021), and Iwasaki et al. (2022), supporting the fourth hypothesis that ownership by a foreigner has an efficacious and consequential association with profit margin, ROA, and Tobin’s Q. Family ownership (FAMOWN) showed mixed results, having negative associations with operating margins and positive ones with financial performance. The result partially supported the fifth hypothesis and conformed to Jadoon et al. (2021). This may be due to mixed opinions about the family stakes in Pakistan. It is generally considered in Pakistan that most firms have been operating with the majority of family members. Family stakes have been incorporated to overcome the negativity of external forces. When family members work as CEOs, top managers, chairpersons, or directors of companies, the pragmatic link is extremely strong (Chu, 2011).

The results of the effect of OWNC (ownership concentration) on top block holders were partially consistent with Anwar and Tabassum (2011), Alkurdi et al. (2021), Martínez-García et al. (2021), and Queiri et al. (2021) and had mixed influence on performing areas. In all the stepwise regressions, the major influence of the concentration was negative, therefore, partially supporting the sixth hypothesis. The coefficient value of BACTIVE (board activeness) fully supported the seventh hypothesis and demonstrated the aligned influence of the board activity on the performance execution as with Vitolla et al. (2020) and Queiri et al. (2021). This result inferred that board activity in the participation in the interim meetings brought fruitful results for the firms. After the separate effects of each phenomenon of the explanatory factors, the interactional effect of board activeness was depicted stepwise in Tables 4–6. The moderating effect of the board activeness with all the ownership stakes demonstrated mixed and differential influence, which partially supported the eighth hypothesis.

## 5. Conclusion

The study was conducted to analyze the effect of state, associated companies, foreign, ownership concentration, and institutional and family ownership on firm execution. The moderating effect of board activeness on the relationship between the structure of corporate ownership and firm execution was also measured. Ownership held by the state, associated concerns, institutions, and foreigners showed consolidated results and fully supported the hypothetical relationships. These ownerships were fully favoring the operating, financial, and stock market performances of the firms and sent the signal to stakeholders to take their ownership stakes accordingly. Family concerns and ownership concentrated in firms demonstrated mixed results. Board activity in the form of interim meetings fully supported the firm performance and code of law, which asked the firms to meet more to discuss and decide about the fortune of the firms positively. The moderating effect of board activity with all the ownership stakes posited differential outcomes for the firms. Most of the interactional influence was negative and not in favor of the firm.

This study is generally supportive for researchers, investors, practitioners, and policymakers. The study provided in-depth information about the stakes of state, associated concerns, foreign, ownership concentration, institutional, and family ownership on firm execution in terms of non-financial firms to practitioners and policymakers that can help them in decision-making. The results of the study are expected to be valuable for investors in their investment dealings, especially concerning ownership by the state, associated concerns, institutions, and foreigners. Specifically, the management of the firm should utilize the state entry in the ownership. Policies being drawn by the firm should be aligned with the rules and regulations of the Pakistani state. As a mentor, the state subsidizes the resources to be utilized by the firm. The more the firm is attached to the state, the safer it is. Second, the study highlighted that associated companies are very kind to the firm. The firm should avail of the buying, selling, and credit services of the associates. Third, the perpetual consistency of the results of the various studies asserts that institutions in the ownership prove to be an angel for the firm not only in providing a monetary cushion but also in resolving agency issues. Management can illuminate its credit darkness by the optimal linkage with the institutions. Fourth, the positivity of the foreign portfolios gives a very clear note that the management of the firm should avail the foreign stake to grasp the foreign markets. Fifth, management needs to take care of the family and concentrated stakes. The role of other stakeholders becomes important at a time of mixed results. Sixth, effective boards eradicate all the hassles in the way of improved performance. They control the negative externality between principles and agents. Seventh, an effective management strategy is demanded at the time of interaction between the board and the stakeholders. Market players exert a very gigantic influence in the Pakistani market. The varied outcome of the interaction of the stakeholders with the board needs to be watched to keep the interest of each one intact. The study is mainly focused on the non-financial sector for a specific time duration, and the results of the study may not be generalized to financial firms. Therefore, a comprehensive study involving financial and non-financial firms can be conducted for better results in future.

## Data availability statement

The raw data supporting the conclusions of this article will be made available by the authors, without undue reservation.

## Author contributions

FY: overall supervision and analysis. RH: consolidating the sections and initial draft. IK: literature review and methodology. ML: review and editing. All authors contributed to the study.
